# Labor trafficking in marijuana production: a hidden epidemic in the shadows of the cannabis industry

**DOI:** 10.3389/fsoc.2023.1244579

**Published:** 2023-12-12

**Authors:** Jaya Prakash, Timothy B. Erickson, Marti MacGibbon, Hanni Stoklosa

**Affiliations:** ^1^Brigham and Women’s Hospital, Boston, MA, United States; ^2^Division of Medical Toxicology, Department of Emergency Medicine, Mass General Brigham, Harvard Medical School, Harvard Humanitarian Initiative, Boston, MA, United States; ^3^U.S. Department of State, Washington, DC, United States; ^4^National Behavioral Health Association of Providers, Washington, DC, United States; ^5^Mentari Human Trafficking Survivor Empowerment Program, New York, NY, United States; ^6^California Consortium of Addiction Programs and Professionals, Sacramento, CA, United States; ^7^Department of Emergency Medicine, Brigham and Women’s Hospital, Harvard Medical School, Heal Trafficking, Boston, MA, United States

**Keywords:** human trafficking, labor trafficking, cannabis, occupational health, forced labor

## Abstract

Labor trafficking in marijuana production remains a concealed epidemic within the expanding cannabis industry. This abstract brings attention to the systemic exploitation of vulnerable individuals engaged in cultivating, harvesting, and processing cannabis. It explores the factors contributing to labor trafficking, including demand for cheap labor, inadequate regulation, and the vulnerability of the workforce. By compiling published cases, both in peer-reviewed literature and the media, this perspective piece investigates the extent of health issues experienced by labor-trafficked victims. These include chronic pain from repetitive tasks, respiratory problems due to exposure to pesticides and other toxic substances, musculoskeletal injuries, malnutrition, and mental health disorders stemming from trauma and extreme stress. Additionally, this perspective article examines the factors contributing to poor health outcomes of labor-trafficked victims, including hazardous working conditions, lack of access to healthcare, and physical and psychological abuse. Addressing the health challenges faced by labor-trafficked victims in the cannabis industry requires multidimensional solutions: awareness among healthcare providers, comprehensive medical services, and mental health support. Furthermore, collaborative efforts among government agencies, healthcare providers, labor organizations, and the cannabis industry are essential in preventing trafficking and addressing the health disparities faced by labor-trafficked victims.

## Introduction

Human trafficking is defined by United States (U.S.) law as a category of criminal offenses that use force, fraud, or coercion to compel an individual to engage in commercial sex acts or labor against their will (U.S. Department of State, 2022). The U.S. largely recognizes two forms of human trafficking: forced labor and sex trafficking. Forced labor or labor trafficking is defined as “the range of activities involved when a person uses force, fraud, or coercion to exploit the labor or services of another person” (U.S. Department of State, 2022). Sex trafficking is defined as “the range of activities involved when a trafficker uses force, fraud, or coercion to compel another person to engage in a commercial sex act or causes a child to engage in a commercial sex act” (U.S. Department of State, 2022). Historically, public interest in the anti-trafficking movement has centered upon the trafficking of women and children across the boundaries (Ramiz et al., 2020). This stereotype has since metastasized into a publicly accepted definition of the ‘ideal’ or ‘innocent’ trafficking victim, which is an inaccurate representation of the intersecting systems of disempowerment and marginalization that the trafficking industry preys upon. Namely, the population of trafficked persons experiencing exploitation in illicit or partially illicit industries by their particularly vulnerable circumstances has been overshadowed (Einbond et al., 2023). This perspective will focus on labor trafficking in the cannabis industry, particularly in the setting of medical marijuana having been legalized in 38 states as of July 2022 and recreational marijuana having been legalized in 19 states (World Population Review, 2023).

The National Broadcasting Company (NBC) recently reported on a cannabis farm in Southern California that was illegally growing cannabis and profiting off forced labor (News, 2022). The victims had lost employment during COVID, and, feeling desperate for income, relocated to Southern California for work. They were uncompensated for their labor, housed in squalid trailers, and endured threats to their physical safety (Edward, 2021; News, 2022). In addition to exploitation for labor within the cannabis industry, some victims are also exploited for sex, or experience sexual assault. One example of exploitation in the setting of cannabis production was publicized through a 2016 article describing the prevalence of sexual violence perpetrated upon ‘trimmers’ (i.e., individuals responsible for trimming and preparing cannabis plants for sale) within Humboldt County, a cannabis-producing hotspot in Northern California. Specifically, the Domestic Violence Services for Humboldt County experienced an almost 80% increase in all volume over four years, and this increased volume aligns with the experience of other Northern California counties involved with cannabis production. Despite the individuality of each narrative, several overarching connections to sex trafficking were clear: coerced sexual acts, abduction, violence, unbalanced power dynamics, and misleading employment offers (HuffPost, 2016).

### Why does this problem exist?

Recent estimates on marijuana sales and consumption offer a glimpse into the expansion of the cannabis industry. In some U.S. states where cannabis is still deemed illegal, cannabis is the most frequently used federally illegal drug. Over 48.2 million Americans reported having used the substance at least once in 2019 (Walter, 2016). Further compounding the harms posed by this rapidly expanding industry is the lack of research on the cannabis industry and its utilization of labor exploitation. This lack of awareness impedes the detection and identification of potentially trafficked persons within the cannabis industry. Moreover, “cannabis growers who are victims of trafficking are often dealt with as offenders,” especially given that the reductive “police framing of growers as drug criminals, with the accompanying perfunctory consideration of modern slavery fits nicely with traditional, binary concepts of slavery” (Ramiz et al., 2020). Aside from the adverse effects of law enforcement categorizing trafficked persons as criminals, there is also the question of ulterior motives that may fuel “policing for profit,” which has been previously associated with the government’s war on drugs. For example, in 2015 alone, over 2,000 crisis calls were placed to the Humboldt Domestic Violence Services, an increase that was largely attributed to the region’s growing affiliation with the cannabis industry. Law enforcement seized over $2 million of assets over that year, which contributed both a finder’s fee and a cut of confiscated funds to the marijuana eradication team (Walter, 2016). The concern is that these financial incentives may be fueling tunnel vision that focuses on eradicating marijuana at the expense of observing and identifying human exploitation in the setting of these criminal offenses [Migrant Rights Centre Ireland (MRCI), 2014]. Finally, the ever-changing legal landscape of cannabis means that even in places with elements of cannabis industry legality, there is limited regulation, lack of protection of worker rights and inadequate whistleblower policies, creating conditions that allow traffickers to exploit people for labor (Allain et al., 2013; Beckman et al., 2023).

### What are the health implications of this problem?

The inclusion of survivor narratives and disaggregated research regarding the experiences of trafficked individuals within the context of cannabis manufacturing is scant. This perspective article compiles the few published cases (both in peer-reviewed journals and the media) that exist within the fund of literature on the subject. Several overarching themes are identified that exist for this vulnerable sub-population of individuals that warrant further investigation to aid in the identification and response of this public health problem [Migrant Rights Centre Ireland (MRCI), 2014; HuffPost, 2016; News, 2017, 2020, 2022; Ramiz et al., 2020; Williams and Yu, 2020; Edward, 2021; Parfitt, 2022].

Across all narratives, there are clear, persistent needs and stressors (e.g., income, stability, family circumstances, national/international conflict) that drive persons to be trafficked into the cannabis industry. Semi-structured interviews with labor trafficking survivors demonstrate that political persecution and financial need not only drove immigrants from their home countries but also pressured their search for employment opportunities (Ramiz et al., 2020; Williams and Yu, 2020). Individuals were misled by a third party (e.g., smuggler, recruiter) to believe the employment opportunity–which for the most part involved travel to a different region or country–aligned with their prior work history (Edward, 2021). For victims who were traveling to a different country without documentation, many were smuggled across international borders and housed by their employers, repayment for which was tallied into victims’ work demands in the form of debt bondage (Ramiz et al., 2020; Parfitt, 2022; U.S. Department of State, 2022). Some victims were not even aware as to which country they had ended up in Parfitt (2022). Therefore, for migrant workers, there were the added challenges of an unfamiliar community, lack of connections, and language barriers (Williams and Yu, 2020; Edward, 2021). A similar driver of financial need complicated by the misleading nature of employment opportunities existed among individuals who experienced sex trafficking within the cannabis industry [Migrant Rights Centre Ireland (MRCI), 2014; HuffPost, 2016; News, 2020]. In reality, victims were oftentimes under-compensated if compensated at all [Migrant Rights Centre Ireland (MRCI), 2014; HuffPost, 2016; News, 2017, 2020; Ramiz et al., 2020; Williams and Yu, 2020; Edward, 2021; News, 2022; Parfitt, 2022]. Once at their final destination, survivors of all trafficking types were locked in, not permitted to leave or not able to leave because of geographic isolation in extremely remote settings. Lack of ability to leave was reinforced by threats to the trafficked or their family, physical locks, inaccessible exits, and armed security guards (Tbilisi City Court, 2011; Allain et al., 2013; HuffPost, 2016; Ramiz et al., 2020; Parfitt, 2022).

There are significant hazards that survivors faced through out labor trafficking in the cannabis production process. Both labor and sex trafficking survivors reported threats to their safety and health: “If I try to run away if they found me they would kill me” (Ramiz et al., 2020); “threatened him that he would be very sorry if anything happened to them” (Parfitt, 2022); “threatened to kill her, freeze her body, and throw her to the animals if he ever found out” (HuffPost, 2016). The threats of violence were not empty. Violence was inflicted during the survivors’ journey and within the context of their workdays (HuffPost, 2016; News, 2020; Ramiz et al., 2020). Housing conditions among labor-trafficked survivors were universally inadequate, unsanitary, and dangerous: “inside the barn, the heat was stifling” (Parfitt, 2022); “brought us food only once a week” (Parfitt, 2022); “slept on a blanket on the floor” (Ramiz et al., 2020; Searchlight New Mexico, 2023). They lacked access to food, water, and hygiene resources resulting in health-related issues such as exhaustion and hospitalization for “exposure” (News, 2017; Ramiz et al., 2020; Edward, 2021; Parfitt, 2022). The exorbitant work demands and abhorrent living conditions were found to be fatal for several individuals (Williams and Yu, 2020). Post-traumatic stress disorder and other forms of psychological distress were also seem among labor trafficking victims exploited in the cannabis industry (P. v. The Chief Superintendent of The Garda National Immigration Bureau & ors, 2015; Kárníková, 2023). Finally--though this has not been explored in the context of labor trafficking within the cannabis industry--there is prior literature speaking to substance use among trafficked people and agricultural workers that is introduced and/or exploited by work supervisors to maintain control (Bletzer, 2004; Koegler et al., 2022). Supervisors may then deduct the cost of these substances from workers’ modest wages thereby rendering them unable to leave the exploitative situation via a tactic known as manipulated indebtedness (Bletzer, 2004).

### What are the occupational hazards of this problem?

The occupational risks of labor-trafficked individuals involved in cannabis cultivation has yet to be fully investigated. With that said, evidence to date has separately identified the occupational health and safety for workers employed in the cannabis industry as well as for migrant workers employed in agricultural settings. This section will review both the occupational risks for both groups that, based on the previously described case stories, are most likely experienced by labor-trafficked cannabis workers.

Given the increasing demand for cannabis products, there has been a call to evaluate the potential hazards associated with cannabis harvesting and processing tasks. In response, the Health Hazard Evaluation Program through the Centers for Disease Control and Prevention, as well as the National Institute for Occupational Safety and Health, assessed work practices, air samples, surface samples, demonstrations, and employee concerns at an outdoor cannabis farm to better understand workers’ occupational health. The report identified highly repetitive and forceful work (e.g., trimming) performed by workers that increased their risk of musculoskeletal issues, potential for skin and oral exposure to THC, and airborne exposure to Actinobacteria and fungus that can increase the risk of respiratory disease (Couch, 2017). Potential exposures faced by workers include “particulate matter, organic dust, bioaerosols, pollen, allergens, volatile organic compounds, pesticides, and ergonomic hazards” (Simpson, 2020). For indoor cannabis growing operations, the biggest concerns seem to be related to mold, fungus, and moisture issues, which can be associated with severe respiratory conditions (e.g., asthma, shortness of breath, respiratory infections, allergy exacerbation, and reactive airway disease). In fact, “these exposures are significantly increased and are consistent with those that would be experienced in mold remediation activities” (Martyny et al., 2013).

Migrant workers are particularly vulnerable to labor trafficking given their employment in ‘3-D’ jobs “dirty, dangerous, and demanding (sometimes degrading or demeaning),” which keeps them “often hidden from or invisible to the public eye and form public policy” (Moyce and Schenker, 2018). As a result, they are more frequently subjected to harmful occupational exposures that result in a 15% higher likelihood of a work-related fatal injury when compared to native-born workers. For those migrant workers in the agricultural industry, common environmental hazards include temperature, pesticides, chemicals, physical hazards, occupational demands, and lack of safety control (Moyce and Schenker, 2018; Stoklosa et al., 2020). Heat exhaustion is a common concern given the high ambient temperature of agricultural work in the setting of fast-paced tasks, lack of breaks, and inordinately long work days (Moyce and Schenker, 2018; Stoklosa et al., 2020). Workers often lack personal protective equipment when working with pesticides, which can result in pesticide poisoning (Stoklosa et al., 2020). Unyielding demands of manual labor create opportunities for musculoskeletal injuries: “exposure to hazardous equipment, crush injuries, repetitive motions, and falls” (Moyce and Schenker, 2018). Not suprisingly, measures to prevent occupational hazards and healthcare resources to address clinical problems are not readily available to those being trafficked, which disproportionately increases the health risks faced by this population (Moyce and Schenker, 2018; Stoklosa et al., 2020).

## Discussion

Hopefully, this commentary will serve as a call for interdisciplinary action across all major stake-holding disciplines to address human trafficking more effectively and intentionally in cannabis production. Given the dearth of evidence-based guidance within this area, there is a strong need for both quantitative and qualitative research. Public health practitioners and academic researchers are well-positioned to shed light on the experiences, needs, risk factors, and health outcomes of this population to effectively inform screening, identification, resource coordination, care management, and prevention efforts across these settings. As law enforcement may often be the first actors called to the scene of forced labor or sexual exploitation in the setting of criminal drug offenses, police officers should be well-trained in this issue. In particular, they can utilize trauma-informed practices in gauging potentially exploitative circumstances, investigating the workings of traffickers, and connecting survivors to health as well as social resources [Migrant Rights Centre Ireland (MRCI), 2014]. From a clinical perspective, though our specific knowledge around this form of exploitation is limited, many of the health and safety concerns suggested through these narratives align with evidence-based indicators and the health needs of labor-trafficked individuals in the larger context of anti-trafficking literature. Patients may present with injuries following occupational hazards, evidence of undocumented status, persistent monitoring by a third party, psychological trauma, malnutrition, sexually transmitted infections, and injury via interpersonal violence (Coppola and Cantwell, 2016; Couch, 2017 DeCicco et al., 2023). Trafficking survivors may exhibit a broad variety of clinical signals including: “use or abuse of recreational drugs or alcohol; impaired judgment; feeling or demonstration of emotional exhaustion; feeling depersonalized or demonstration of lack of self-confidence; a constant feeling of fear, nervousness or anxiety; or abdominal pain, headaches, fatigue, dizzy spells, back pain, pelvic pain, and sexually transmitted infections” (Coppola and Cantwell, 2016; DeCicco et al., 2023). Health systems should be be prepared to connect those experiencing labor trafficking in the cannabis industry to resources and educate their staff on this form of exploitation (Baldwin et al., 2023).

## Data availability statement

The original contributions presented in the study are included in the article/supplementary material, further inquiries can be directed to the corresponding author.

## Author contributions

JP: Investigation, Writing – original draft, Writing – review & editing. HS: Conceptualization, Investigation, Resources, Supervision, Validation, Writing – review & editing. TE: Conceptualization, Resources, Supervision, Validation, Writing – review & editing. MM: Conceptualization, Investigation, Resources, Writing – review & editing. All authors contributed to the article and approved the submitted version.
